# Supplementary material for: PRAMEL7 and CUL2 decrease NuRD stability to establish ground-state pluripotency
Source: EMBO Rep. 2024 Feb 8;25(3):1453–68. doi: 10.1038/s44319-024-00083-z (PMC10933316; doi:10.1038/s44319-024-00083-z)
Supplement: Supplementary file 1 — Appendix [file 44319_2024_83_MOESM1_ESM.pdf]

Appendix

PRAMEL7 and CUL2 decrease NuRD stability to establish ground-state pluripotency

Rupasinghe, Bersaglieri *et al.*

Table of content **Page**

Appendix Figure S1. PRAMEL7-CUL2 axis contrasts the repression of genes associated with NuRD complex (related to Fig. 6). 2

Appendix Figure S1

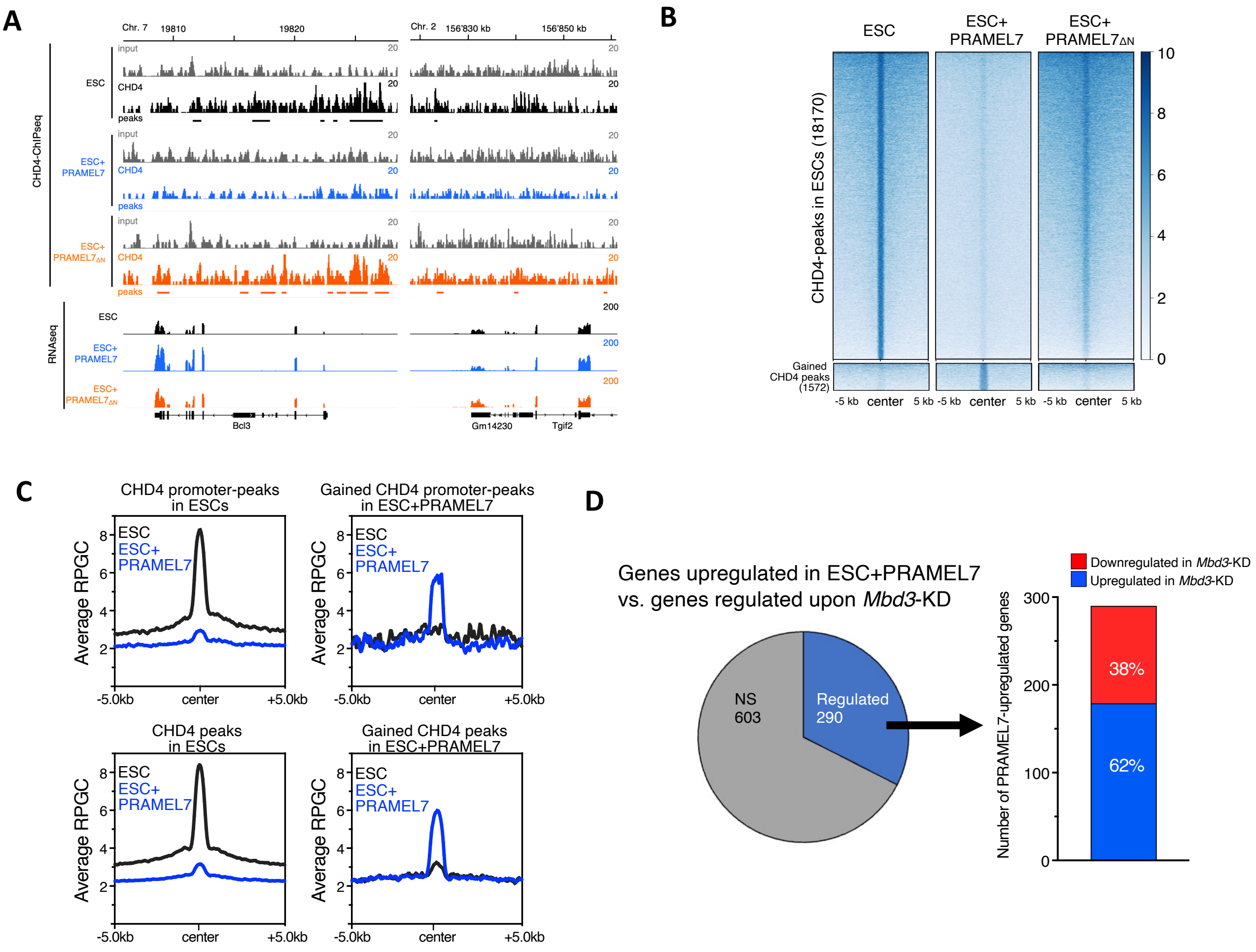

Appendix Figure S1

PRAMEL7-CUL2 axis contrasts the repression of genes associated with NuRD complex

A. Representative images showing CHD4-association in ESCs (ChIPseq) and gene expression (RNAseq) in parental ESCs, ESC+H/F-PRAMEL7$_{WT}$, and ESC+H/F-PRAMEL7$_{\Delta N}$ of two upregulated PRAMEL7$_{Cul2}$- genes with CHD4-bound promoters.

B. Heatmap showing all CHD4 peaks detected in parental ESCs and the corresponding signals in ESC+H/F-PRAMEL7$_{WT}$ and ESC+H/F-PRAMEL7$_{\Delta N}$.

C. Average density plots of ChIPseq read counts of CHD4 peaks in parental ESCs and gained CHD4 peaks in ESC+PRAMEL7 at ± 5 Kb from CHAD4 peak summits. Data are represented for total CHD4 peaks and CHD4 peaks within promoters.

D. Quantifications of upregulated PRAMEL7 genes that are also upregulated in *Mbd3*-KD ESC+serum. RNAseq data are from (Luo *et al.*, 2015).
