# Supplementary figures and images for: PRAMEL7 and CUL2 decrease NuRD stability to establish ground-state pluripotency
Source: EMBO Rep. 2024 Feb 8;25(3):1453–68. doi: 10.1038/s44319-024-00083-z (PMC10933316; doi:10.1038/s44319-024-00083-z)

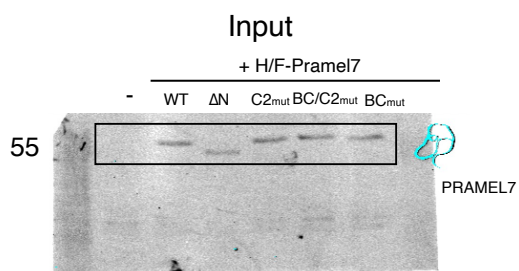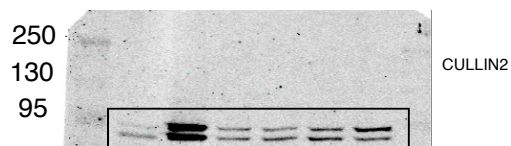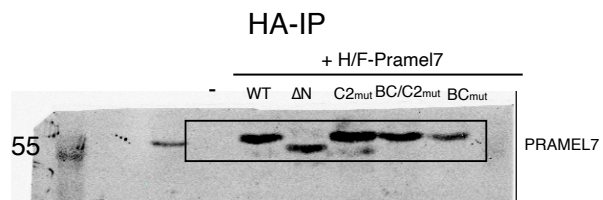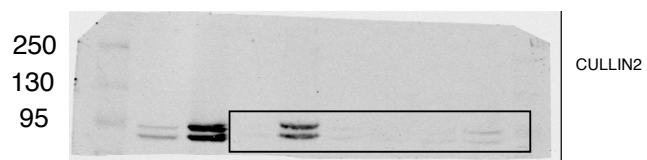

Supplement: Supplementary file 11 — Source Data Fig. 1 [file 44319_2024_83_MOESM11_ESM.zip › Figure1/1C.pdf]

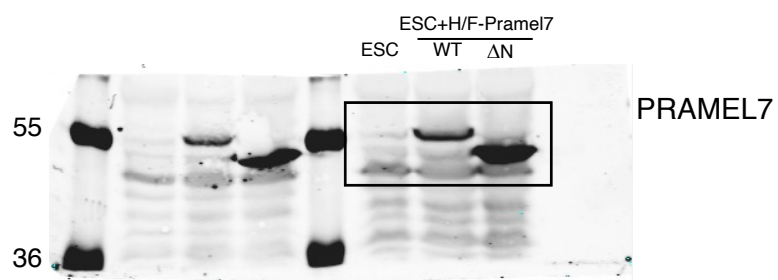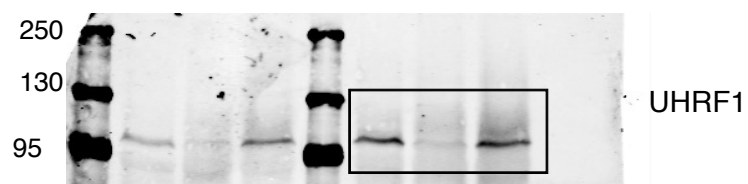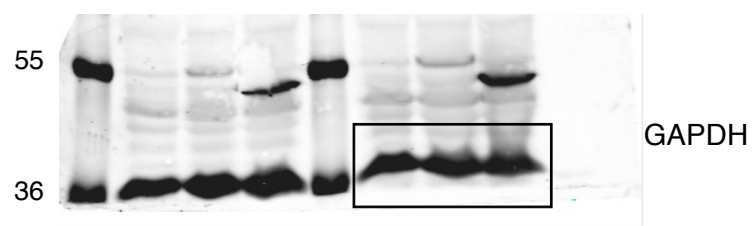

Supplement: Supplementary file 11 — Source Data Fig. 1 [file 44319_2024_83_MOESM11_ESM.zip › Figure1/1F.pdf]

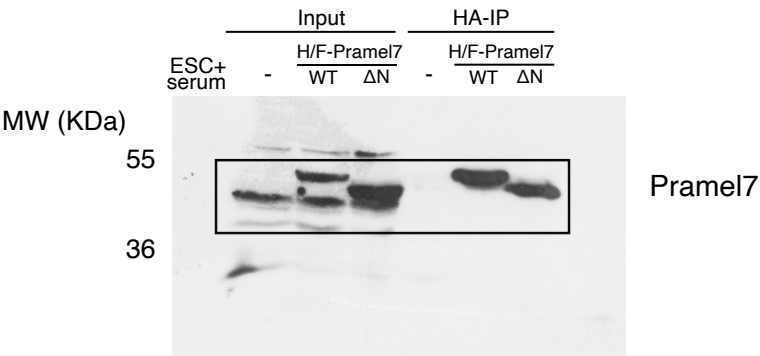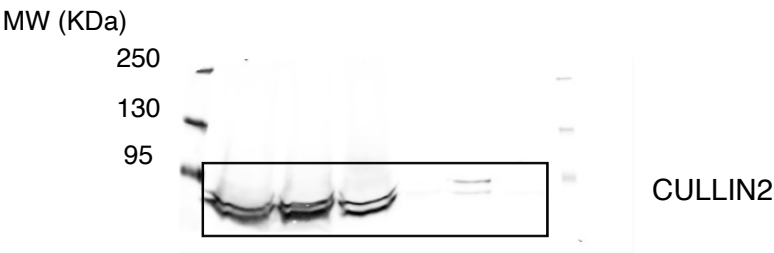

Supplement: Supplementary file 11 — Source Data Fig. 1 [file 44319_2024_83_MOESM11_ESM.zip › Figure1/1E.pdf]

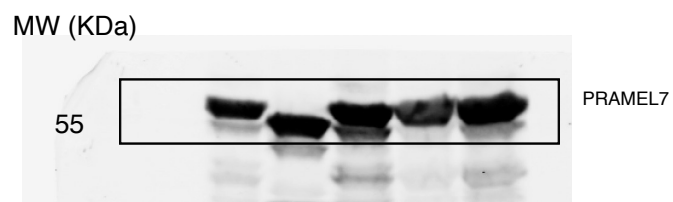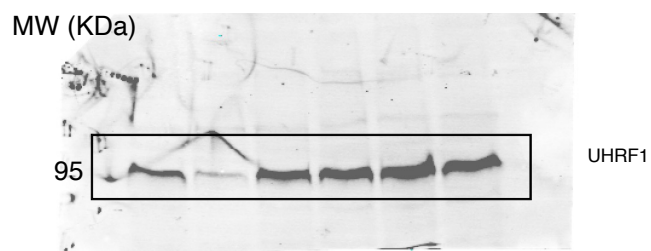

Supplement: Supplementary file 11 — Source Data Fig. 1 [file 44319_2024_83_MOESM11_ESM.zip › Figure1/1D.pdf]

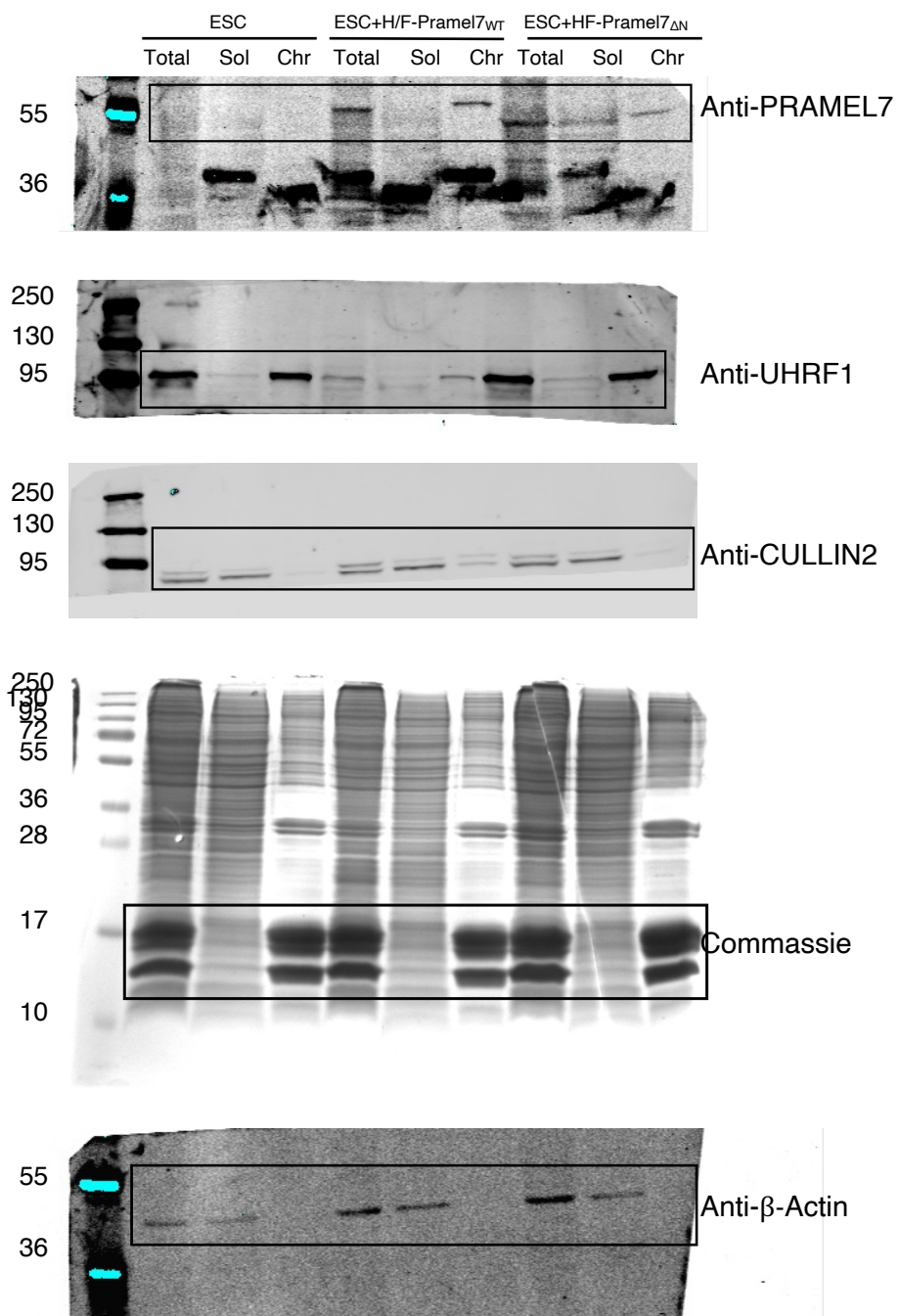

Supplement: Supplementary file 13 — Source Data Fig. 4 [file 44319_2024_83_MOESM13_ESM.zip › Figure4/4A.pdf]

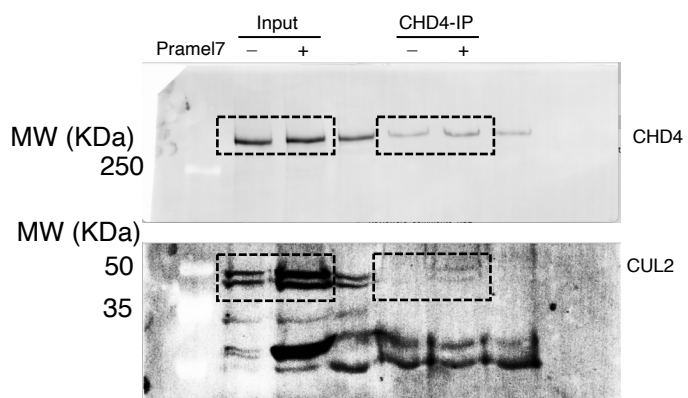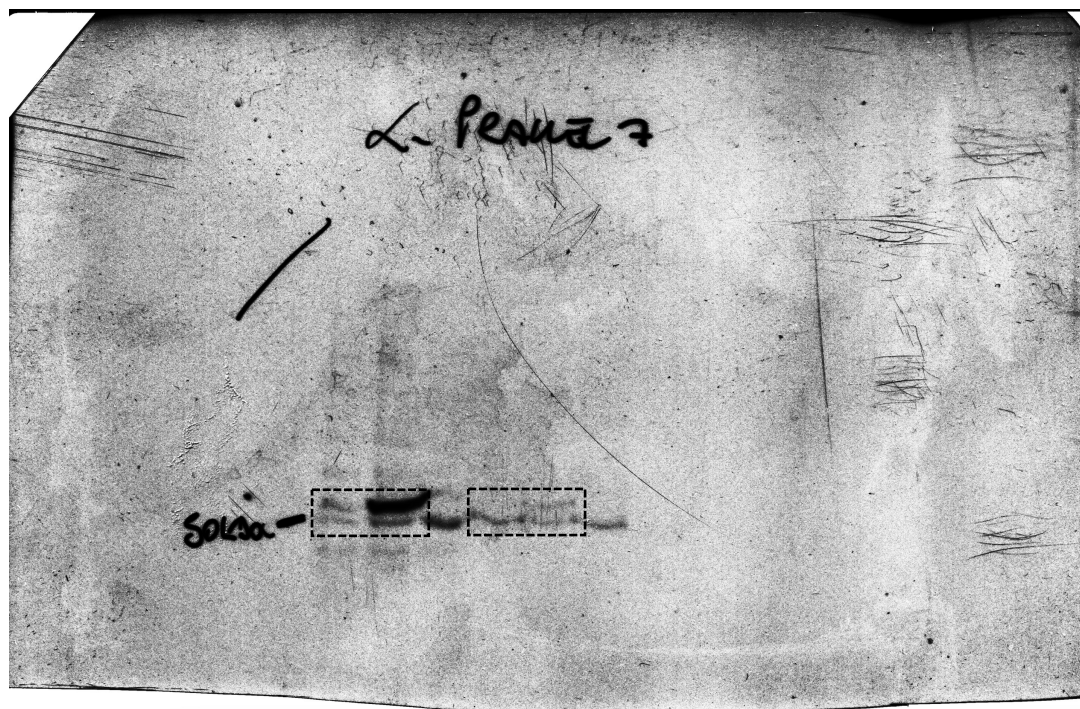

Supplement: Supplementary file 15 — Source Data Fig. 6 [file 44319_2024_83_MOESM15_ESM.zip › Figure6/6A.pdf]
